# Identification of novel polyethylene-degrading fungi from South African landfill soils: *Arthrographis kalrae, Lecanicillium coprophilum,* and* Didymosphaeria variabile*

**DOI:** 10.1007/s10532-025-10170-0

**Published:** 2025-07-31

**Authors:** Nozipho Kheswa, Arun Gokul, Nontembeko Dube

**Affiliations:** 1 Department of Zoology and Entomology, University of the Free State, Private Bag x13, Phuthaditjhaba, 9866 South Africa; 2 Department of Plant Sciences, University of the Free State, Private Bag x13, Phuthaditjhaba, 9866 South Africa; 3 Department of Zoology and Entomology, University of the Free State, P.O. Box 339, Bloemfontein, 9300 South Africa

**Keywords:** Polyethylene biodegradation, Plastic pollution, Novel fungal species, Environmental microbiology, Solid waste management, Plastic-degrading microorganisms

## Abstract

The persistent inefficiency of landfill operations and plastic waste management in South Africa has intensified environmental contamination, underscoring the urgent need for innovative bioremediation strategies. This study aimed to identify and evaluate fungal isolates from landfill soils for their ability to biodegrade polyethylene (PE), thereby contributing to sustainable plastic waste management solutions. A total of eighteen fungal isolates were recovered from local landfill soils using plastic-enriched soil dilution techniques. These isolates were screened for PE biodegradation by incubating pre-weighed polyethylene strips with each fungal culture for 45 days at ambient temperature. Biodegradation efficiency was assessed through gravimetric weight loss, while structural alterations in the polymer matrix were examined using fourier transform infrared (FTIR) spectroscopy and scanning electron microscopy (SEM). Several isolates demonstrated significant PE degradation, including the novel PE degraders *Arthrographis kalrae* SP5INT, *Lecanicillium coprophilum* SP7MK, and *Didymosphaeria variabile* SP11INT, reported here for the first time. *Penicillium chrysogenum* SP17MK and *Engyodontium album* SP3MK showed the highest degradation rates, achieving over 20% weight loss. FTIR analysis revealed the appearance of carbonyl groups (~ 1700 cm⁻^1^) and a reduction in characteristic PE peaks at 719 and 1472 cm⁻^1^, suggesting oxidative degradation. SEM imaging further confirmed surface erosion and structural disintegration of the polymer, supporting the biochemical evidence of degradation. These findings represent the first report of novel fungal species capable of degrading PE in South African landfill soils and significantly expand the known diversity of plastic-degrading fungi. This work highlights South Africa's emerging role in microbial bioremediation research and provides a foundation for the development of locally relevant, biologically based plastic waste management strategies.

## Introduction

Plastic pollution remains a major global concern, with significant and well-documented environmental impacts. Despite these concerns, global plastic production continues to rise, reaching over 1.9 million tons in 2021, an increase of 4.7% from the previous year (Plastics 2021). Plastics are valued for their versatility, lightweight nature, durability, and cost-effectiveness, making them indispensable across industries (Ncube et al. 2021). Polyethylene (PE) and polypropylene (PP) are widely used in packaging and retail due to their functional properties (Sugii 2008). However, these polymers are highly resistant to biodegradation, leading to long-term environmental accumulation (Sangale et al. 2019). The environmental hazards of PE have become increasingly evident: plastics can persist for decades or centuries, fragmenting into microplastics (< 5 mm) that infiltrate soil and aquatic systems, adsorb heavy metals and organic toxins, and pose ingestion risks to wildlife and humans (Barnes et al. 2009; Lwanga et al. 2017; Wang et al. 2019). These microplastics disrupt soil biodiversity, harm invertebrates like *Enchytraeus crypticus* and *Porcellio scaber* (Lahive et al. 2019; Selonen et al. 2020) and have been linked to reduced microbial diversity and impaired plant growth (Matjašič et al. 2021; Naidoo and Rajkaran 2020). Among the most persistent pollutants is low-density polyethylene (LDPE), a widely used non-biodegradable plastic whose resistance stems from its hydrophobicity and stable carbon–carbon bonds (Jeon et al. 2021; Zhang et al. 2022). Historically, waste disposal relied on practices such as “dilute, bury, or burn,” which left soil, groundwater, and air polluted with persistent organic and inorganic contaminants (Agbeshie et al. 2020; Sigel and Sigel 1992).

To address PE’s resistance, several physical and chemical pretreatment methods have been explored, including using ultraviolet (UV) irradiation, thermal oxidation, and chemical treatments (e.g., nitric acid, ozone, Fenton’s reagent) (Duddu et al. 2015; Moharir and Kumar 2019). These treatments oxidize the polymer surface, introducing functional groups (carbonyl, hydroxyl) that increase hydrophilicity and improve microbial colonization and enzymatic action (Cowan et al. 2022; Duddu et al. 2015). Despite their effectiveness, these methods may produce secondary pollutants (Moharir and Kumar 2019). Consequently, combining physical and chemical pretreatments with microbial biodegradation has been proposed as a synergistic and environmentally friendly strategy to accelerate the breakdown of otherwise highly resistant polymers (Awasthi et al. 2017; Moharir and Kumar 2019).

Biodegradation of LDPE by microorganisms offers a promising and sustainable alternative. Certain microbial species, including fungi, can secrete extracellular oxidative enzymes (e.g., laccases, peroxidases) that break down long-chain hydrocarbons into smaller molecules ultimately mineralized to CO_2_ and H_2_O (Labuzek et al. 2006; Munir et al. 2018; Zhang et al. 2022). Prior studies have shown fungal genera such as *Aspergillus, Penicillium, and Fusarium* spp. colonize plastic surfaces, introducing functional modifications (e.g., carbonyl, hydroxyl, double bonds) detectable by FTIR spectroscopy and considered key markers of biodegradation progression (Cowan et al. 2022; Gong et al. 2023; Munir et al. 2018; Sowmya et al. 2015). Deterioration of LDPE is characterised by the oxidative introduction of functional groups, particularly carbonyl (C = O) and hydroxyl (O–H) groups, which increase hydrophilicity and facilitate microbial enzymatic attack. Subsequent microbial metabolism fragments the long hydrocarbon chains into monomeric, dimeric, and oligomeric by-products that may be assimilated into microbial cell membranes (Cowan et al. 2022). These biochemical changes are routinely monitored using FTIR spectroscopy: for example, reductions in absorbance at characteristic wavenumbers such as 1710–1715 cm⁻^1^ (carbonyl), ~ 1640 cm⁻^1^, and 830–880 cm⁻^1^ (–C = C–) have been reported during fungal degradation of LDPE, confirming progressive oxidation and depolymerization (Hasan et al. 2007). The microbial degradation process typically proceeds through polymer deterioration, fragmentation, assimilation, and mineralization (Cowan et al. 2022; Restrepo-Flórez et al. 2014; Wei and Zimmermann 2017), with outcomes assessed by monitoring changes in functional groups, hydrophobicity, crystallinity, surface topography, mechanical properties, molecular weight distribution, and complete utilization of the polymer (Sen and Raut 2015).

In South Africa, plastic waste management faces additional challenges: the country ranks 11th globally and 3rd in Africa for mismanaged plastic waste (Babayemi et al. 2019; Jambeck et al. 2015). In rural provinces like the Free State, only ~ 11% of waste is formally collected, with over 60% of households relying on informal disposal methods (Board 2018). Despite introducing Africa’s first plastic bag levy in 2003, consumer behaviour has not shifted significantly, and plastic use continues to increase (Dikgang et al. 2012; Sadan and Kock 2020). These challenges underscore the need to investigate local, sustainable solutions. Biodegradation of LDPE by locally adapted fungi offers a promising, low-cost strategy for mitigating plastic accumulation. However, most existing studies have focused on laboratory strains or isolates from outside southern Africa, leaving a significant knowledge gap regarding indigenous fungal species and their biodegradation potential. This study therefore aims to isolate and identify fungal species from landfill soils in the eastern Free State province of South Africa and assess their ability to degrade pretreated LDPE under different growth conditions. By integrating weight loss measurements, pH changes, and FTIR analysis of functional group modifications, this research contributes to developing region-specific strategies for plastic waste management and enhances scientific understanding of fungal biodegradation potential in South African landfill environments.

## Materials and methodology

### Study area and soil sample collection

**Fig. 1:**
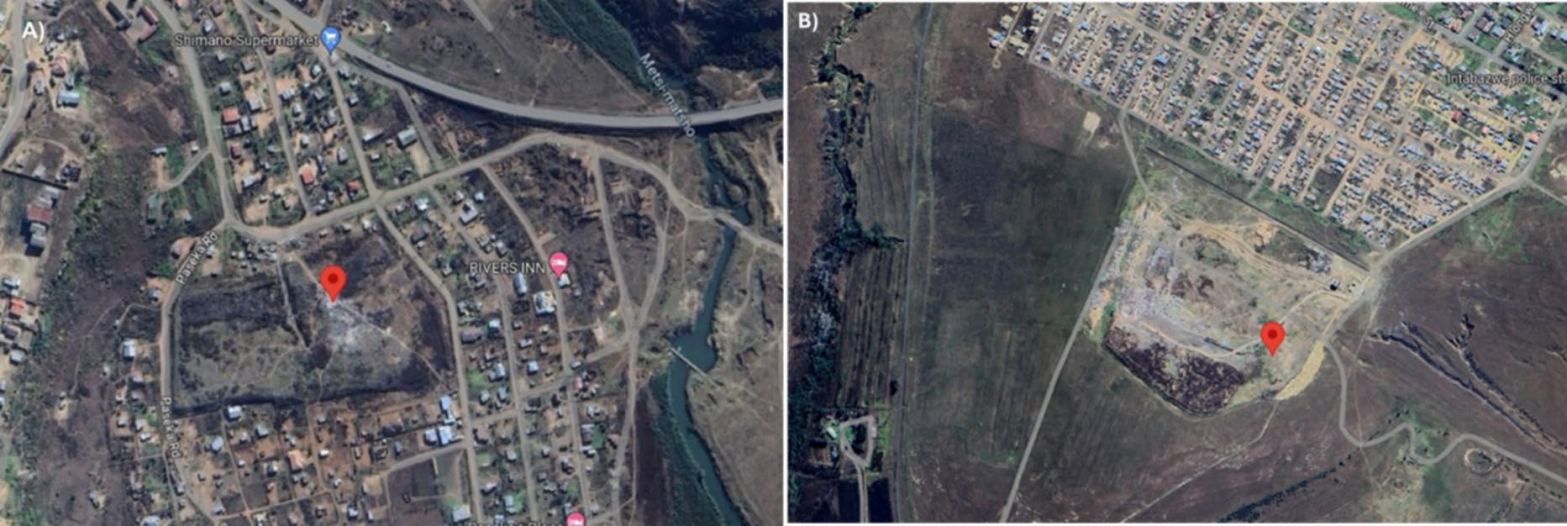
Locations of soil sample collection sites in Maluti-A-Phofung Municipality: **A** Phuthaditjhaba landfill and **B** Harrismith landfill. These sites were selected for fungal isolations aimed at identifying species with potential PE biodegradation capabilities. Image source: Google Earth

This study was conducted in the Maluti-A-Phofung local municipality of the Free State Province, South Africa, and the soil was sampled from the two landfill sites (Harrismith and Phuthaditjhaba; Fig. 1). The landfill sites were used to search for the soil fungal species with plastic bioremediation capability. The soil samples were collected in August 2022. Soil samples were collected by dividing landfill sites into 3 parts (front, middle, and back zones), and 10 soil samples were collected randomly with a clean, dry auger and put in sterile polythene Ziplock bags. Because most of the microbial activity is concentrated near plant roots, additionally, plants growing within the landfill sites were uprooted to collect soil near roots. The soil samples were transported in a cooler box with ice to the laboratory and kept at 4 °C until metal analysis and fungal isolation were conducted.

### Plastic material

Two types of LDPE single-use plastic bags were selected to evaluate the biodegradation potential of local fungal isolates. The first type was a conventional (virgin) LDPE plastic bag manufactured from unprocessed primary polymer. The second type was an unconventional NRCS-approved LDPE bag (IRCS/8087/246977/0043) produced entirely from 100% recycled plastic (non-virgin), representing post-consumer material. Both plastic types are widely used in retail packaging in South Africa and were chosen for their local relevance.

Using a sterile surgical blade, plastic strips measuring 1 cm × 4 cm with an average nominal thickness of approximately 20 μm were prepared for experimental assays. Typical LDPE film has a density of ~ 0.915–0.935 g/cm^3^ and an ultimate tensile strength (UTS) of approximately 23–26 MPa, with high ductility reflected by elongation at break often exceeding 600%. Virgin LDPE generally exhibits higher tensile strength and greater elasticity due to its higher molecular weight and absence of prior degradation, whereas recycled LDPE may contain polymer chain scission, residual additives, and impurities, leading to reduced mechanical strength and lower elongation at break (Hopewell et al. 2009).

### Fungal isolation and screening for potential growth in polymer-enriched media

Isolation of fungal species from the soil was performed using a standardized procedure (Raja et al. 2017). Soil extracts were obtained by shaking 10 g of soil in sterile water; the supernatants were then serially diluted. One millilitre of a 1/100 dilution of the soil solution was plated using the pour plate technique onto five replicates of media, namely mineral salt medium (MSM), containing (in grams per litre of distilled water) 0.5 K_2_HPO_4_ (dipotassium hydrogen phosphate); 0.04 KH_2_PO_4_ (monopotassium phosphate); 0.1 NaCl (sodium chloride); 0.002 CaCl_2_·H_2_O (calcium chloride dihydrate); 0.2 (NH_4_)_2_SO_4_ (ammonium sulphate); 0.02 MgSO_4_·7H_2_O (magnesium sulphate heptahydrate); 0.001 FeSO₄ (iron(II) sulphate); and 20.0 agar. The media were supplemented with antibiotics (chloramphenicol, 0.1; and streptomycin sulphate, 0.55 reagents; and 1% polyethylene solution (Sigma-Aldrich) to selectively isolate fungi with the ability to degrade polymers. The media was autoclaved for 15 min at 121 °C and then cooled to 55 °C before plating the diluted soil solution using the pour plate technique. The plates were incubated at 28 °C for 6 days. The fungal species that were able to grow on a polymer-enriched medium were considered for plastic biodegradation. Pure cultures were produced using plates containing potato dextrose agar (PDA) and Rose Bengal agar media. One-centimetre agar blocks containing fungal isolates were aseptically transferred onto plates with solidified agar media. The plates were incubated at 28 °C until the growth of pure fungal isolates was observed. The isolated fungal species were then preserved in a 20% glycerol solution, prepared by mixing 375 ml of sterile water with 125 ml of 80% glycerol in a 1000 ml sterile bottle. Each fungal isolate was transferred into a sterile screw capped cryogenic vials labelled with date and isolation identification code. For preservation, the fungal isolates were stored in glycerol. This was achieved by adding 8 ml of 20% glycerol to the medium plates containing the fungal isolates. A sterile loop was used to gently scrape the isolates from the medium plate, and the resulting mixture was transferred into sterile, screw-capped cryogenic vials. The vials were stored appropriately at −80 °C.

### Morphological identification of fungal isolates

The isolated fungi were identified to the genus level and to the species level, when possible, based on macromorphology. The colonies were examined for slow or rapid growth, topography (flat, heaped, regularly or irregularly folded), texture (yeast-like, powdery, granular, velvety, or cottony), surface pigmentation and reverse pigmentation, micromorphological (hyphae, macroconidia, microconidia, chlamydospore) and other special fungal structure characteristics using suitable media, slide cultures, and the most updated keys for identifications. Micrographs of the fungal isolates, the hyaline mycelium, were stained using lactophenol and cotton blue solution. The stained specimens were observed under the light microscope (Eclipse E200LED MV R, Nikon, Tokyo, Japan) equipped with a 5-megapixel microscope camera (Delta Pix Invenio 5SIII, Denmark). For identification, the microphotographs were taken under 100X magnification. The captured photographs were processed by DeltaPix InSight software (Version 6.0). The morphological identification of fungal isolates was verified using molecular tools (described in the characterisation section below).

### Genetic confirmation of the fungal isolates

The fungal isolates were confirmed and characterised using molecular tools by an accredited laboratory (Inqaba Biotechnical Industries (Pty) Ltd., in Pretoria, South Africa). Specifically, 100 mg of fungal mycelium was collected from the solid Rose Bengal medium after 6 days of growth. This mycelium was then used for DNA extraction using the Quick-DNA Fungal/Bacterial Miniprep Kit (Zymo Research). The polymerase chain reaction (PCR) reaction (10–30 μl) was carried out using 1.5 mM MgCl2, 0.25 mM of each dNTP, NEB OneTaq 2X MasterMix with Standard Buffer, 1 U/μl Taq DNA polymerase, 10 pmol of each Internal Transcribed Spacer (ITS) gene primer, and 50 ng DNA template PCR amplification was carried out using the ITS1 primer: 5’-TCCGTAGGTGAACCTGCGG-3’ and ITS4 primer: 5’-TCCTCCGCTTATTGATATGC-3’ for all fungal isolates. The PCR (gradient thermocycler, Applied Biosystem, USA) was programmed with an initial denaturation at 94 °C for 5 min, followed by 35 cycles of denaturation at 94 °C for 30 s, annealing at 50 °C for 30 s, extension at 68 °C for 1 min, and a final extension at 68 °C for 10 min. The PCR products were separated on a 1% agarose gel (CSL-AG500) with 1X TAE buffer as a negative control and a 100 bp DNA ladder. The amplified bands were eluted from the agarose gel using the Zymo Research ZR-96 DNA Sequencing Clean-up kit as per the instruction manual and sequenced in the forward and reverse direction. The obtained sequences were viewed on Finch TV and curated to create contigs with CLC Bio Main Workbench. BLASTn analysis was performed on the NCBI website to determine if a sequence in the database matches the query sequence above a certain threshold (99% query coverage; 99% identity). All fungal species' ITS gene sequences were submitted to GenBank and accessioned. Phylogenetic analyses were carried out on the ITS sequences saved from the GenBank using MEGA software 7.0. The neighbouring joining method was used to deduce the evolutionary history of all the fungal isolates by calculating the evolutionary distance by applying the p-distance method and evaluating the branches with 1000 bootstrap replications.

### Biodegradation test of LDPE by fungal isolates using broth medium method

The fungal species were screened for polyethylene biodegradation potential following the standardized method described by Sangale et al. (2019). each fungal isolate was obtained from six-day-old pure cultures grown in Sabouraud’s broth, and 1 ml of the resulting fungal suspension was used to inoculate the test medium. Conidial spore counts were determined using a Neubauer haemocytometer (0.1 mm depth), and conidial concentration was calculated through serial dilutions (Lacey 1997). The final conidial concentration achieved was 9.52 × 10^2^ conidia/ml. The fungal isolates were screened for their potential to degrade polyethylene by regularly shaking the broth medium inoculum with LDPE strips at room temperature, in triplicates. After 45 days of continuous shaking, polythene degradation was assessed using percent reduction or loss in weight (%WL).

Polythene carry bags were procured from the local markets in QwaQwa in the Free State. With the aid of a sharp surgical blade, 1 × 4 cm strips of the LDPE were made. As per the protocol of Sharma and Sharma (2004), pretreatment of the LDPE strips was carried out to remove the additive, if any. Before subjecting fungal infection, LDPE strips were weighed using a precision balance and sterilised, followed by UV treatment for 15–20 min in laminar air flow. To each sterile LB broth test tube, three LDPE strips were transferred aseptically. One millilitre of 2-day-old fungal culture was transferred to each test tube (three test tubes/isolate). After inoculation, all the cultures were placed on an orbital rotary shaker at the speed of 140 ± 20 rpm at room temperature for a period of 45 days. After 45 days of incubation, the screening of the polythene degrading fungal isolates was carried out based on the percent weight loss of the LDPE strips.

### Change in pH

Changes in pH were evaluated to assess any metabolic activity of the fungal isolates in broth medium, as metabolism shown by microbial cells may greatly support the evidence of degradation (Khan et al. 2023). The pH of each fungal suspension and control was measured before and after 45 days of screening during the study. The pH was measured using a calibrated digital pH meter.

### Biodegradation test of LDPE by fungal isolates using agar medium method

**Fig. 2:**
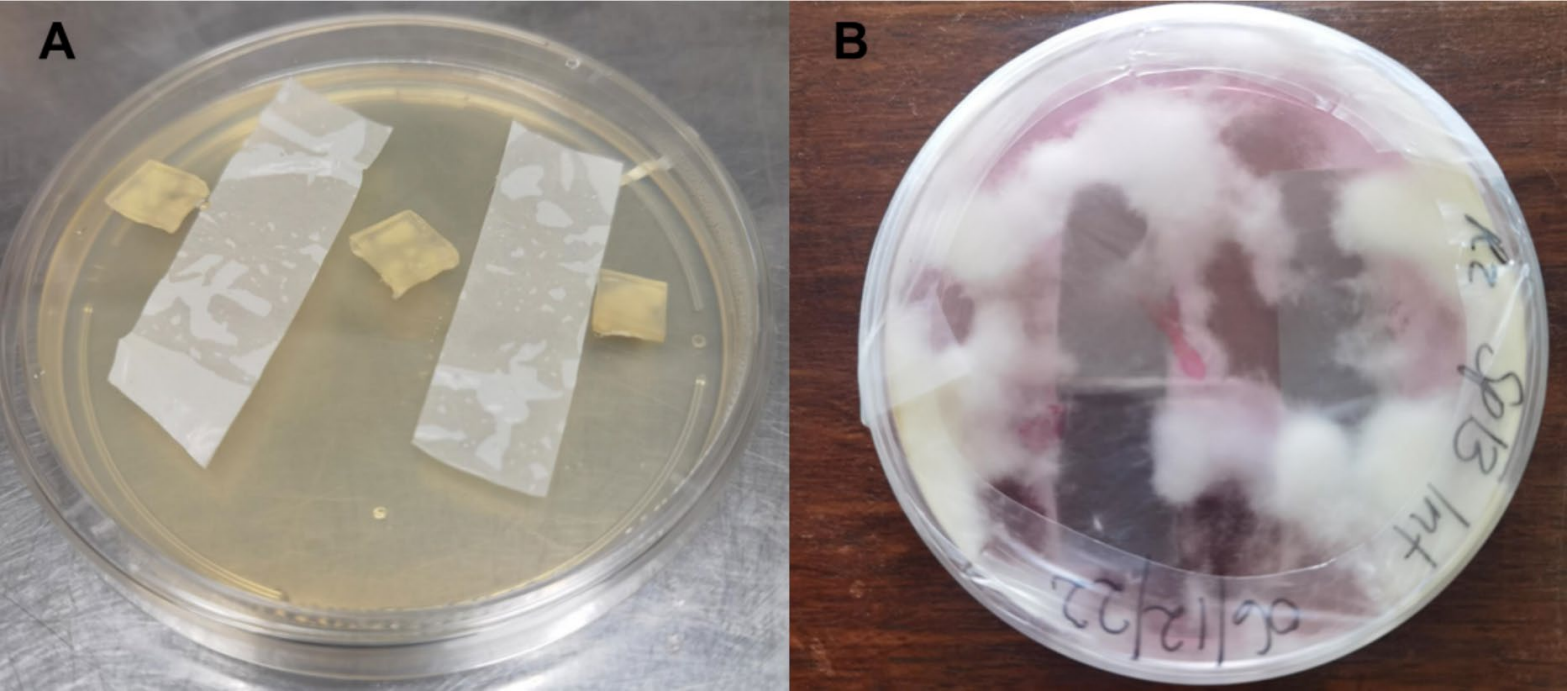
Polyethylene degradation by fungal isolates incubated in 90 mm Petri dishes at room temperature. **A** Introduction of fungal isolates into the agar medium with LDPE strips placed on the surface, showing the setup at the beginning of incubation. **B** Extensive fungal growth observed after 30 days, with hyphal networks covering the PE surface and surrounding agar, indicating active colonization and potential biodegradation

Fungal isolates from landfill soil were used to screen for plastic biodegradation on a solid agar medium in four repetitions (Fig. 2). The solid media were supplemented with antibiotics (chloramphenicol, 0.1; and streptomycin sulphate, 0.55 reagents) (Munir et al. 2018). LDPE strips were cut into 1 cm × 4 cm pieces and aseptically applied to the media surface. Two agar plugs (5 mm) of fully fungal-growing colonies were placed on each side of the LDPE strips. Cultures were incubated at room temperature (28 ± 2 °C) for 45 days. Media plates without agar plug fungus were used as a control to confirm the degradation of LDPE strips. After incubation, the strips were removed from the culture using tweezers and rinsed with 70% ethanol and sterile distilled water. The strips were air-dried at the ambient temperature for 24 h (Munir et al. 2018).

### Screening of LDPE-degrading fungal isolate based on % weight loss

At the end of the 45-day incubation period, the degraded LDPE strips were aseptically removed from each test tube and petri dish, transferred to clean petri dishes, and washed once with absolute alcohol, followed by two rinses with tap water. After washing, all the LDPE strips were dried in an oven overnight at room temperature. The dried LDPE strips were weighed, and the percent weight loss was calculated using the formula:

1 $$ {\text{Weight}}\;{\text{loss }}\left( \% \right) = \frac{Initial\;weight - Final\;weight}{{Initial\;weight }} \times {1}00 $$

where initial weight is the average fresh weight (mg) and final weight is the average final weight after 45 days.

To check the reproducibility of the results, fungal isolates were used for the repetition of the polythene degradation assay. During the repetition assay, both pretreated and untreated LDPE strips (1 × 4 cm) were used, and the assay was performed in a 250-ml flask. All the cultures (with LDPE strips) along with the control (LDPE strips + LB broth) were shaken on a rotary shaker at room temperature. After 45 days of incubation, percent weight loss was calculated.

### Confirmation of the fungal isolate LDPE bioremediation

#### Fourier transform infrared spectroscopy-attenuated total reflection analysis

The changes in the structure of LDPE after the incubation with fungal isolates were determined using Fourier transform infrared (FTIR)-attenuated total reflection (ATR) analyses. After 45 days of shaking and incubation of treated plastic strips with fungal isolates and distilled water (control), strips were placed in separate sterile Petri dishes (90 mm diameter) and rinsed with distilled water, and they were left to air dry under a laminar flow for 24 h. The LDPE strips and those of controls were then analysed in a Perkin Elmar Spectrum 100 FTIR spectrophotometer. FTIR spectra of the film were obtained by placing the PE strip on the spot of the FTIR accessory and slowly pressing. Spectra were recorded in the 4000—650 cm⁻^1^ with a 4 cm⁻^1^ resolution range. The new appearances and disappearances of carbonyl and methyl groups, which changed to methylene, were observed.

Indices such as keto and carbonyl bonds, ester double bonds, and hydroxyl were used to measure the level of degradation, following established methods (Albertsson et al. 1987; Samat et al. 2023).

The carbonyl index measured the amount of carbonyl groups.

2 $$ {\text{Keto}}\;{\text{carbonyl}}\;{\text{index}} = \frac{{{\text{I1715}}}}{{{\text{I1465}}}} $$

3 $$ {\text{Ester}}\;{\text{carbonyl}}\;{\text{index}} = \frac{{{\text{I1740}}}}{{{\text{I1465}}}} $$

Double bond index assessed the concentration of PE double bonds, and

4 $$ {\text{Double}}\;{\text{bond}}\;{\text{index}} = \frac{{{\text{I1640}}}}{{{\text{I1465}}}} $$

Hydroxyl index was used to assess the oxidation process of PE and is useful for rating the degree of degradation.

5 $$ {\text{Hydroxyl}}\;{\text{index}} = \frac{{{\text{I3435}}}}{{{\text{I1465}}}} $$

where the spectra bands are at 1715 cm⁻^1^, 1740 cm⁻^1^, 1465 cm⁻^1^, and 1640 cm⁻^1^, they indicate keto and ester carbonyl, hydroxyl, double bond, and methylene bands, respectively.

### Scanning electron microscopy analysis

The scanning electron microscopy (SEM) was used to assess the morphology of a sample. It uses electron beams, which were produced in the microscope; the beams got projected through the microscope column to the sample on the stage. When the electron of the beam met the particles of the samples, the electrons spread out and travel in a different direction. Unique SEM detectors were then used to interpret the movement of the surface particles. SEM detected the dispersion of polymer matrix filler particles, phase separation in polymer blends, and visualisation of the interaction between compound elements. For SEM analysis, sample preparations were carried out using a previously described method (Kyaw et al. 2012), followed by chromium coating before taking images. Coating was done to create a conducive PE surface. The SEM (Tescan VEGA 3 SEM) was used to check for any surface changes of LDPE after treatment (Pramila and Ramesh 2011). Images of treated and untreated LDPE strips after 45 days of shaking in LB media and incubating in agar media for 45 days were obtained using SEM analysis.

### Statistical analyses

Weight loss data were analysed using two-way analysis of variance (ANOVA), with fungal isolate and LDPE type (virgin vs. non-virgin) specified as fixed factors, including their interaction term (isolate and type). Similarly, changes in pH measured before and after incubation were evaluated using two-way ANOVA, considering fungal isolate and time (before vs. after incubation) as fixed factors and their interaction (isolate and time). Where significant effects were detected, post hoc comparisons among means were conducted using Duncan’s multiple range test. All statistical analyses were performed using SPSS version 20.0 (IBM Corp., USA), based on three independent biological replicates (n = 3) per experimental condition. Data visualization and graph preparation were carried out in GraphPad Prism 6 (GraphPad Inc., San Diego, USA). Statistical significance was accepted at *p* < 0.05.

## Results

### Isolation and screening of fungal isolates with ability to degrade polymers

**Fig. 3:**
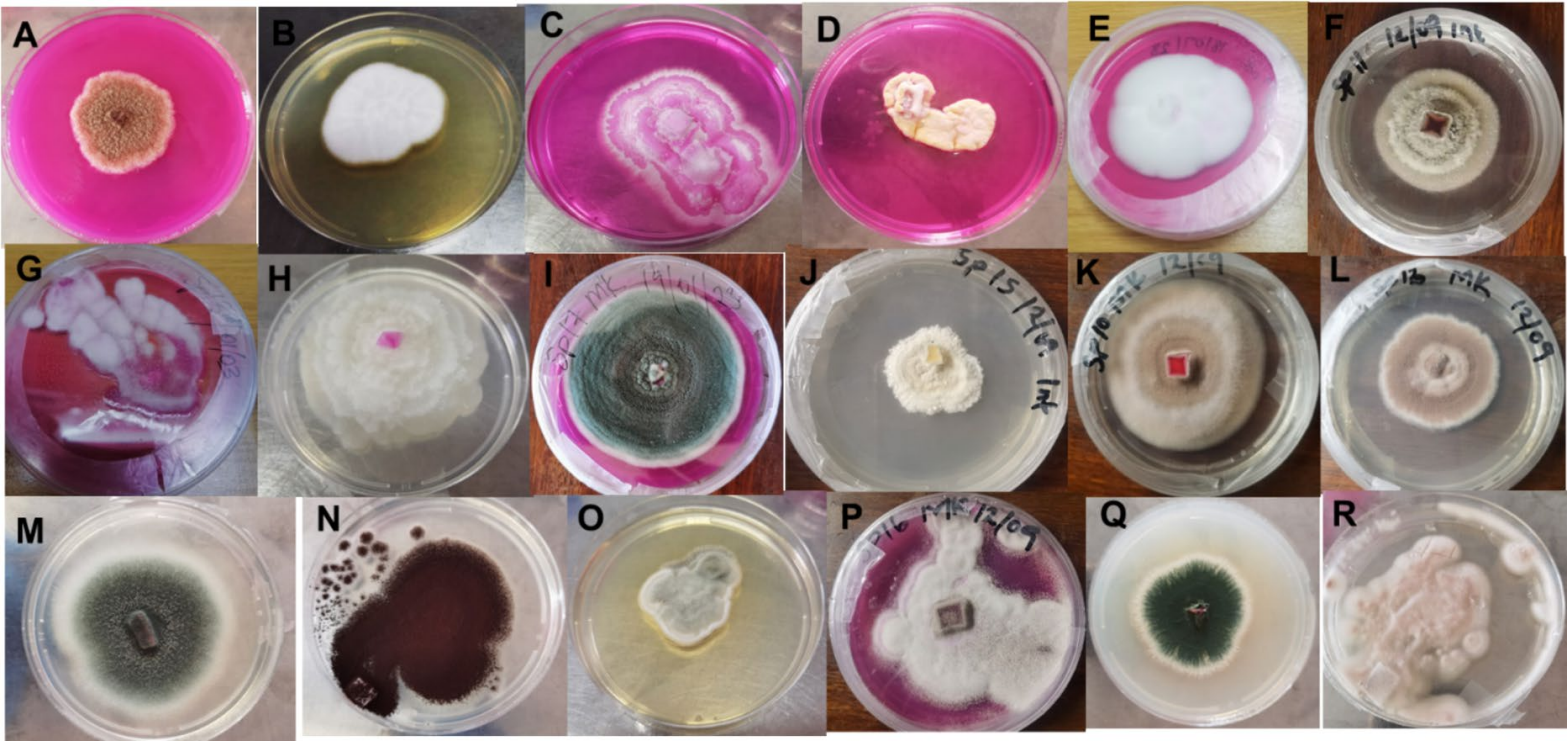
Representative cultures of fungal isolates recovered from landfill site soils and maintained on Rose Bengal and potato dextrose agar (PDA) media. These cultures were preserved for further screening and morphological identification. Panels A–I show the nine isolates that exhibited robust growth in polymer-enriched mineral salt medium: **A** SP2INT, **B** SP3MK, **C** SP5INT, **D** SP6MK, **E** SP7MK, **F** SP11INT, **G** SP13INT, **H** SP17INT, and **I** SP17MK. Panels J–R show the remaining isolates that did not demonstrate growth under polymer-enriched conditions: **J** SP15INT, **K** SP10MK, **L** SP13MK, **M** SP4INT, **N** SP2MK, **O** SP18INT, **P** SP16MK, **Q** SP9INT, and **R** SP18MK

Eighteen fungal isolates were recovered from soil samples collected at the Harrismith and Phuthaditjhaba landfill sites (Fig. 3). Of these, nine isolates (SP2INT, SP3MK, SP5INT, SP6MK, SP7MK, SP11INT, SP13INT, SP17INT, and SP17MK), exhibited robust growth on polymer-enriched mineral salt medium (Fig. 3A–I), suggesting their potential ability to utilize LDPE as a sole carbon source. Based on this preliminary screening, these nine isolates were selected for further assessment of their biodegradation potential. Gravimetric analysis subsequently demonstrated that only five isolates (SP3MK, SP5INT, SP7MK, SP11INT, and SP17MK) produced a significant reduction in LDPE strip weight following incubation, indicating active degradation of the polymer substrate. These five isolates were therefore chosen for additional characterization, including analysis of chemical modifications by FTIR spectroscopy and assessment of surface morphological changes by scanning electron microscopy (SEM), to further validate and elucidate their biodegradation activity.

### Morphological identification of the fungal isolates

**Fig. 4:**
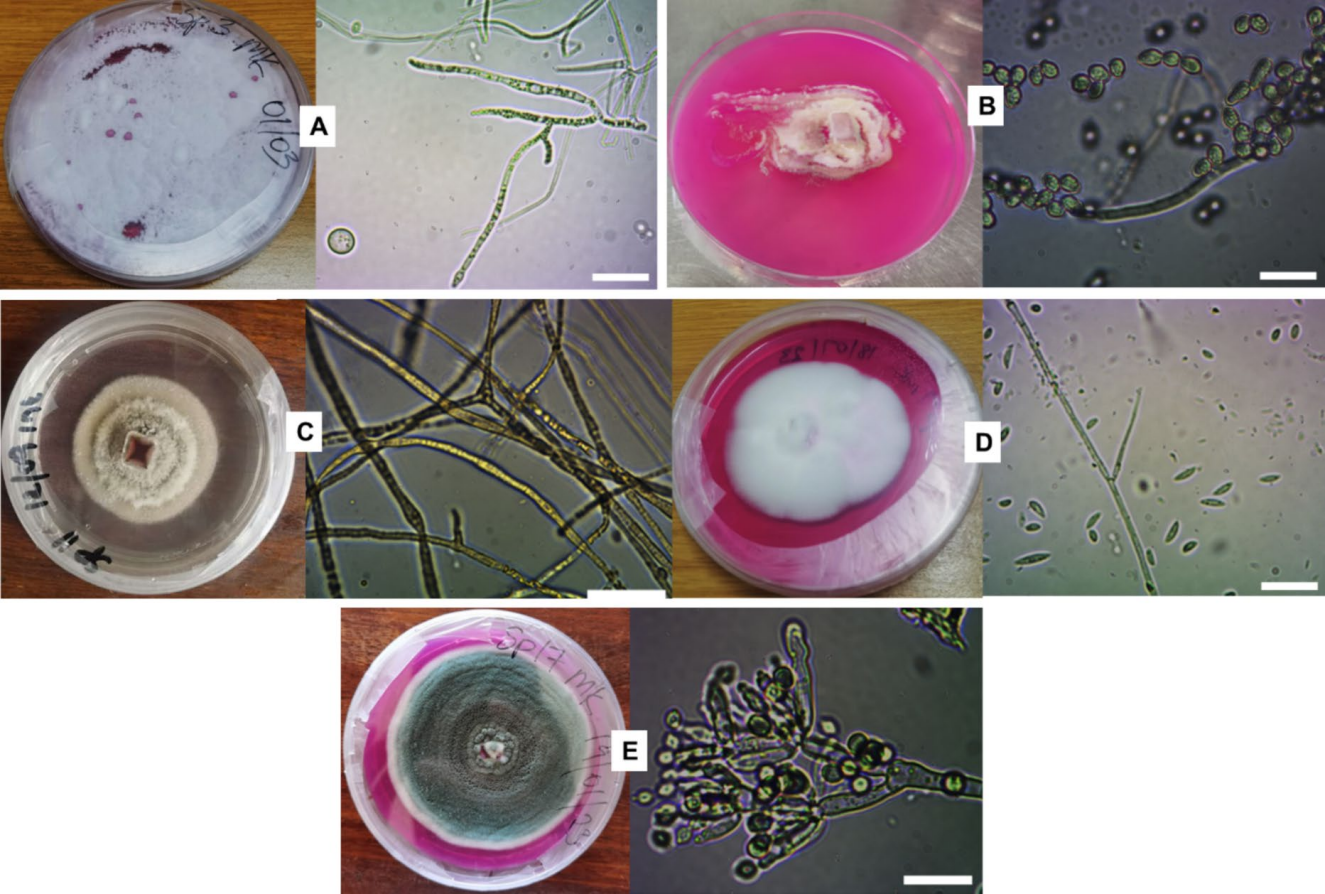
Macro- and micromorphological characteristics of five fungal isolates exhibiting polymer biodegradation potential, isolated from landfill site soil and incubated for 4 days at room temperature. Panels show both macroscopic colony morphology and microscopic features observed under bright field microscopy (scale: 5 μm). **A** SP3MK; **B** SP5INT; **C** SP11INT; **D** SP7MK; and **E** SP17MK. Macroscopic features were documented on agar plates, while microscopic features were visualized to assess spore structure and hyphal characteristics

The morphological and microscopic features of the fungal isolates are shown in Fig. 4 and summarized in Table 1. Overall, the isolates displayed diverse colony colours and textures, ranging from white powdery growth (SP3MK) to dense, cotton-like colonies (SP7MK) and layered morphologies (SP11INT). Microscopically, conidial structures varied from spherical (SP3MK) to elongated, oval forms (SP7MK), reflecting morphological diversity even among isolates with similar biodegradation potential. Molecular identification (Table 2) supported these observations: SP3MK was identified as *Engyodontium* spp., SP17MK as *Penicillium* spp., and the three novel candidates SP5INT, SP7MK, and SP11INT matched *Arthrographis* spp., *Beauveria* spp., and *Didymosphaeria* spp., respectively. Importantly, while SP3MK and SP17MK belong to genera previously reported for plastic degradation, SP5INT, SP7MK, and SP11INT represent taxa not yet documented as polyethylene degraders, highlighting them as promising novel isolates for further study.

**Table 1:** Morphological and microscopic characteristics of fungal isolates recovered from landfill soils

Isolate code	Colony colour/appearance	Colony texture/shape	Microscopic features
SP3MK	White, powdery	Visible conidial development	Non-septate hyphae; spherical conidia, Resembling *Beauveria* spp.
SP5INT	Cream-colored, slow growing	Large, green-pigmented conidia in short chains	Matches *Arthrographis kalrae* morphology
SP7MK	Dense, cotton-like	Oval, elongated conidia	Resembling *Beauveria* spp.
SP11INT	Olive-grey centre; layered	Highly branched hyphal networks	Conforms to *Didymosphaeria variabile* features
SP17MK	Green with whitish margins	Brush-like conidial heads	Typical of *Penicillium* spp.

**Table 2:** List of fungal species isolated from soil samples collected at local landfill sites. Molecular identification was conducted using primer pairs ITS1/ITS4. The table includes the length of the sequenced DNA fragments (in base pairs, bp) and the percentage identity with the closest matching sequences in the NCBI database

Fungus species	Primer	Length (bp)	Identity	Identity
*Penicillium chrysogenum* (SP17MK)	ITS1/4	439	100	MF077262.1
*Engyodontium album* (SP3MK)	ITS1/4	600	99.83	KJ767113.1
*Lecanicillium coprophilum* (Sp7MK)	ITS1/4	577	100	OM491183.1
*Arthrographis kalrae* (SP5INT)	ITS1/4	600	99.82	AB506810.1
*Didymosphaeria variabile* (SP11INT)	ITS1/4	606	99.83	KX869961.1

### Molecular identification of the fungal isolates

**Fig. 5:**
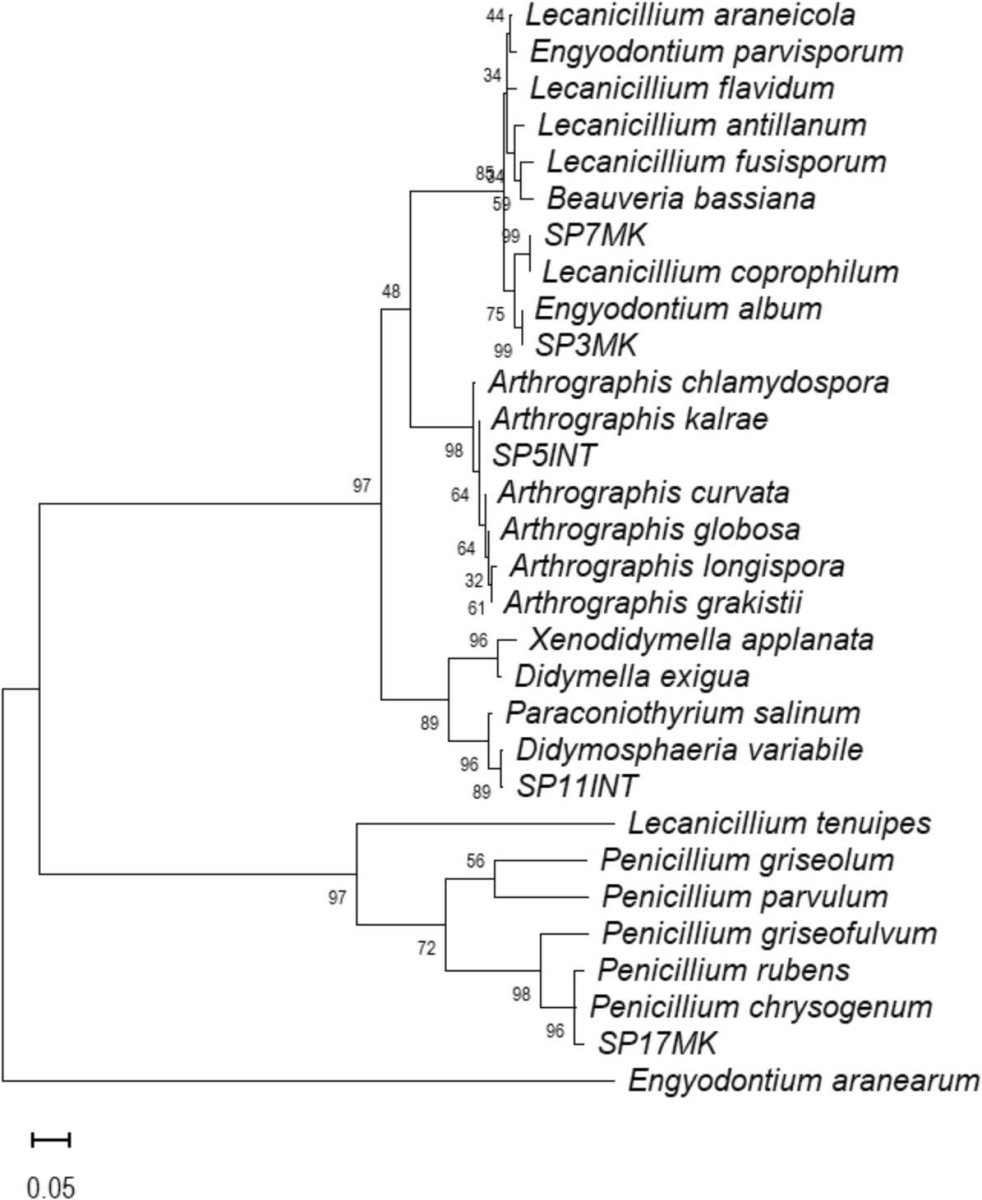
Maximum likelihood phylogenetic trees of fungal isolates based on internal transcribed spacer (ITS) gene sequences. Phylogenetic analyses were performed using MEGA 7.0 software, with bootstrap values (based on 1000 replicates) shown at corresponding branches

The ITS sequence of isolate SP3MK matched *Engydontium album* with 99.83% similarity (GenBank accession no. KJ767115.1) (Table 2). Although its morphology initially suggested affiliation with *Beauveria* spp. due to its elongated, oval-shaped conidia and dense cottony growth, highlighting the limitations of relying solely on morphological characteristics for genus-level identification. Isolate SP5INT showed 99.82% sequence similarity to *Arthrographis kalrae* (accession no. AB506810.1), thereby confirming its identity through molecular data (Fig. 5). Similarly, SP7MK exhibited 100% sequence identity with *Lecanicillium coprophilum* (accession no. OM491183.1), validating its classification. Isolate SP11INT was identified as *Didymosphaeria variabile (*Fig. 5*)*, based on 99.83% sequence similarity with accession no. KX869961.1. Lastly, SP17MK was phylogenetically grouped with *Penicillium chrysogenum*, supported by a bootstrap value of 78%, which, although relatively low, indicated a likely affiliation with this species and suggested possible intra-genus divergence within the *Penicillium* lineage (Fig. 5). Overall, the molecular data reinforced the morphological observations for most isolates, providing a robust foundation for further investigation of their biodegradation potential. Notably, three isolates SP5INT, SP7MK, and SP11INT currently lack reported data on their ability to degrade plastic, highlighting them as novel candidates for further exploration.

### Fungal growth and surface colonization on LDPE strips

**Fig. 6:**
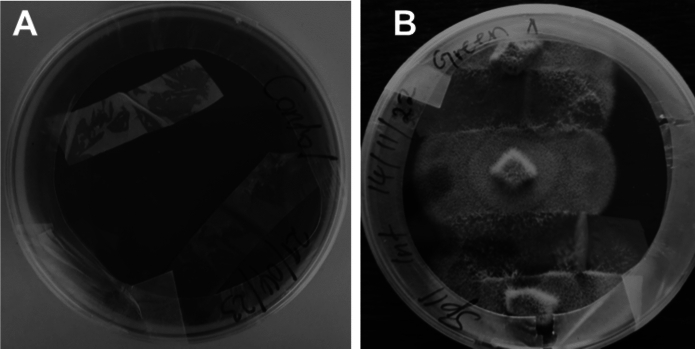
Fungal growth and attachment of mycelium to LDPE strips. **A** Control plate containing LDPE strips without fungal exposure; **B** LDPE strips exposed to fungal isolate *Didymosphaeria variabile* SP11INT. These observations highlight the physical interaction between fungal mycelia and LDPE surfaces during prolonged exposure

The ability of fungal isolates to colonize LDPE strips was assessed after 45 days of incubation (Table 3; Fig. 6). Control plates showed no mycelial growth, confirming the absence of contamination or spontaneous colonization (Fig. 6A). Among the isolates, *Didymosphaeria variabile* (SP11INT) displayed the most extensive colonization, covering ~ 64.5% of the LDPE surface. *Penicillium chrysogenum* (SP17MK) also showed substantial growth, colonizing approximately 45%. In contrast, *Arthrographis kalrae* (SP5INT) exhibited minimal colonization (~ 12.5%), suggesting lower initial surface adhesion (Table 3). Moderate colonization was observed for *Engyodontium album* (SP3MK) and *Lecanicillium coprophilum* (SP7MK), each covering ~ 25% of the surface. All isolates demonstrated statistically significant ability to adhere to and grow on LDPE (Table 3).

**Table 3:** Polyethylene degradation ability of fungal isolates after 45 days of incubation on solid medium. The level of degradation was categorized as high, medium, or low based on visual assessment and extent of fungal colonization and interaction with the PE surface

Isolates	Growth %	Overall potential (low, high moderate)	
		Virgin LDPE	nonvirgin LDPE
**Control**			
*Engyodontium album* (SP3MK)	25	Low	High
*Arthrographis kalrae* (SP5INT)	12.5	Low	High
*Lecanicillium coprophilum* (SP7MK)	25	Low	Moderate
*Didymosphaeria variabile* (SP11INT)	62.5	Moderate	High
*Penicillium chrysogenum* (SP17MK)	45	High	High

^a^Degree of biodegradation potential (low, moderate, high) was estimated based on weight loss resultant after incubation

### Weight loss of LDPE on solid media

**Fig. 7:**
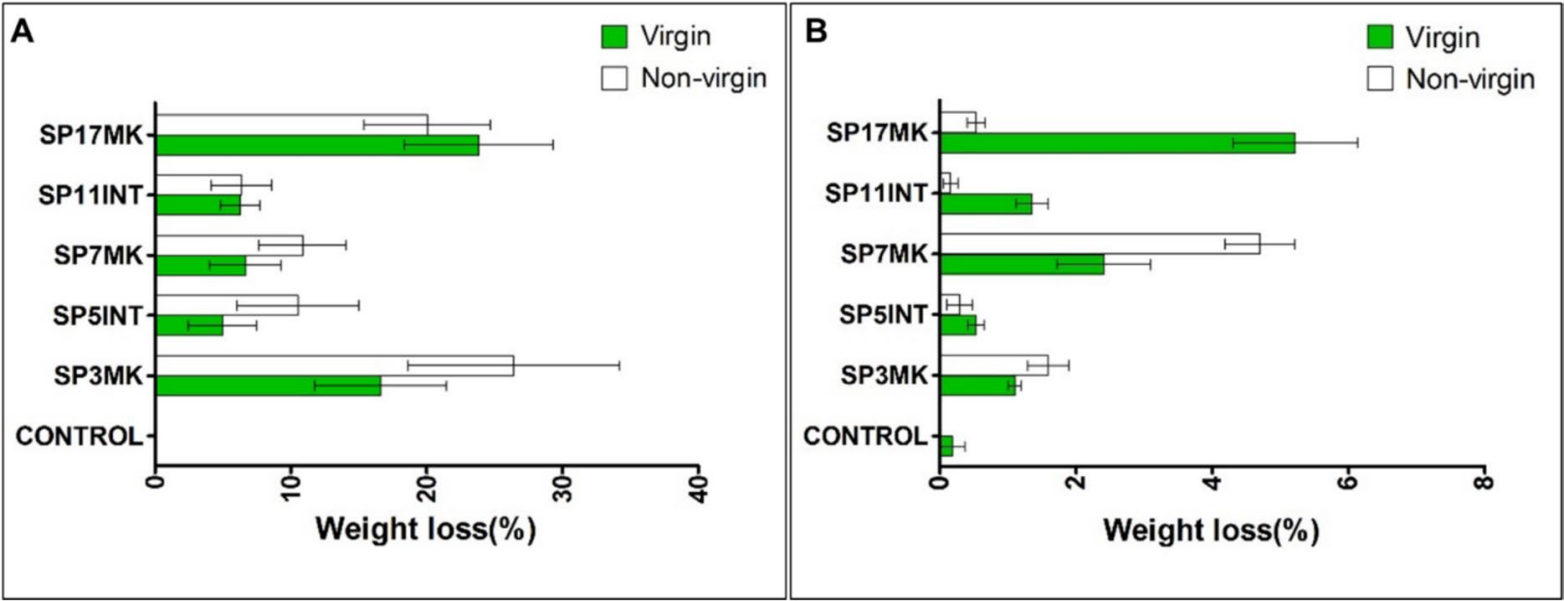
Mean weight loss percentages (± SE) of LDPE strips after 45 days of incubation with selected fungal isolates. The isolates evaluated for their PE biodegradation potential include SP3MK (*Engyodontium album*), SP5INT (*Arthrographis kalrae*), SP7MK (*Lecanicillium coprophilum*), and SP17MK (*Penicillium chrysogenum*). Weight loss was assessed across different conditions: **A** virgin and nonvirgin LDPE on solid medium; and **B** virgin and nonvirgin LDPE on broth medium. These measurements reflect the relative efficiency of each fungal isolate in degrading PE under both nutrient-limited and liquid culture conditions

Weight reduction of LDPE strips after 45 days on solid medium confirmed biodegradation potential (Fig. 7A). Compared to the control, which showed negligible weight change, several isolates caused substantial weight loss. On virgin LDPE, the highest reduction was recorded for SP17MK (24%), followed by SP3MK (17%). SP7MK, SP11INT, and SP5INT achieved lower but measurable reductions (7%, 6%, and 5%, respectively). For nonvirgin LDPE, SP3MK caused the greatest weight loss (26%), followed by SP17MK (20%). SP7MK and SP5INT both achieved ~ 11%, while SP11INT reached 6%. Statistical analysis confirmed significant differences between isolates (F = 9.993; df = 5; *p* < 0.05). There were no differences between LDPE types (virgin vs. nonvirgin; F = 1.348; df = 1; *p* > 0.05). Notably, SP7MK and SP5INT not previously documented as LDPE degraders exceeded 10% weight loss on nonvirgin LDPE, highlighting their promising degradation capacity. Across conditions, SP17MK and SP3MK consistently outperformed other isolates.

### Weight loss of LDPE in broth media

In broth medium, weight loss was generally lower compared to solid media (maximum ~ 5%; Fig. 7B). Despite this, isolates still demonstrated significant biodegradation relative to the control (F = 37.969; df = 5; *p* < 0.05).

SP17MK again achieved the highest weight loss on virgin LDPE (5%), while SP3MK and SP7MK also reduced weight modestly (≤ 3%). On nonvirgin LDPE, the reductions were smaller, and overall virgin LDPE strips lost significantly more weight than nonvirgin strips (F = 11.543; df = 5; *p* < 0.05). The solid media provided more favourable conditions for fungal colonization and enzymatic action on LDPE.

### Comparative biodegradation potential of isolates

The SP17MK and SP3MK isolates emerged as the most effective degraders across both LDPE types and culture conditions, consistently achieving higher weight loss and stronger surface colonization compared to the control and other isolates. Importantly, SP5INT and SP7MK isolates not previously associated with LDPE biodegradation demonstrated moderate colonization and notable weight reduction, especially on nonvirgin LDPE in solid medium (> 10% weight loss).

### The pH changes during biodegradation

**Fig. 8:**
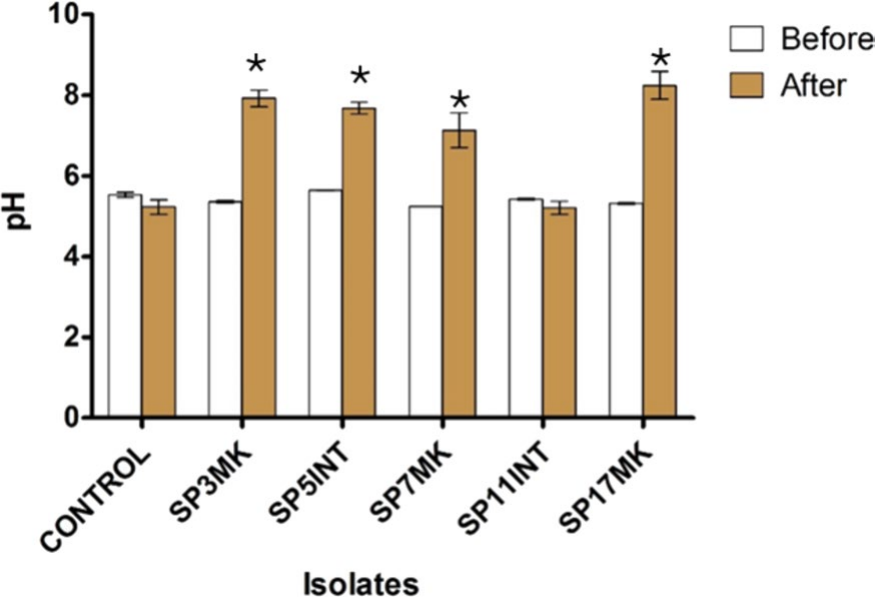
Mean pH values (± SE) recorded before and after 45 days of LDPE strip exposure to fungal isolates in broth medium. Asterisks (*) indicate statistically significant differences between initial and final pH values among the fungal treatments (*p* < 0.05), suggesting metabolic activity and potential involvement in LDPE degradation

The use of broth growth medium enabled the measurement of pH changes during biodegradation. The pH was measured before and after 45 days of exposure to selected fungal isolates in LDPE (Fig. 8). The medium's initial pH value before LDPE inoculation was 5.6, and it remained constant after 45 days of treatment. The control showed a slight decrease from 5.6 to 5.2. The initial pH values for the selected isolates were: SP3MK (5.4), SP5INT (5.7), SP7MK (5.2), SP11INT (5.4), and SP17MK (5.3). After 45 days of incubation, significant pH changes (*p* < 0.05) were recorded for most of these isolates. The pH increased to 7.9 for SP3MK, 7.7 for SP5INT, 7.13 for SP7MK, and 8.2 for SP17MK. In contrast, SP11INT showed minimal change, with a slight decrease from 5.4 to 5.2. Notably, the isolates that induced substantial pH increases, such as SP17MK and SP3MK, also caused significant LDPE weight loss, while SP11INT, with minimal pH change, showed limited biodegradation activity.

### Confirmation of the ability of fungal isolates to biodegrade polyethylene

#### Fourier transform infrared spectroscopy-attenuated total reflection (FTIR)

**Fig. 9:**
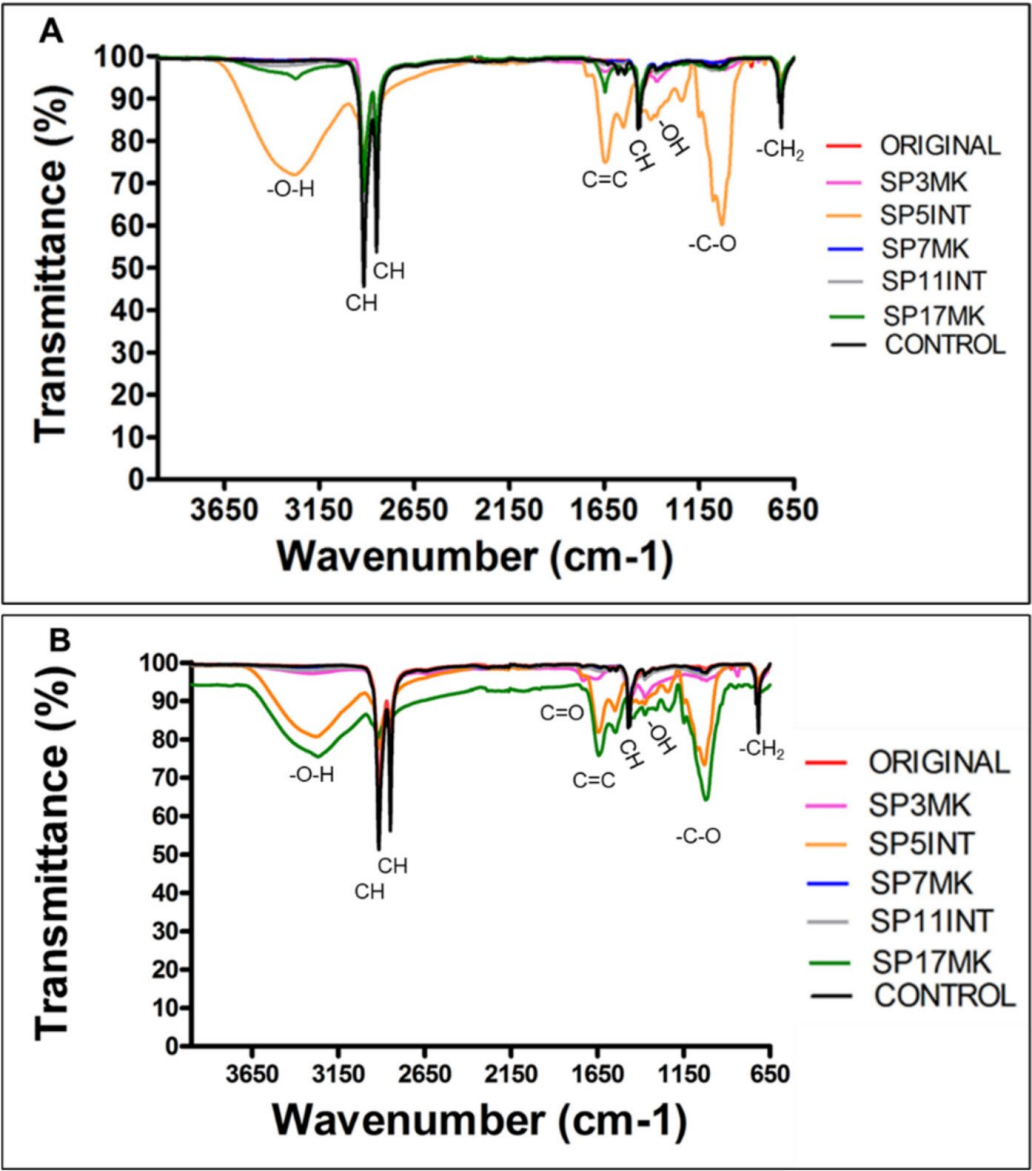
FTIR spectra of LDPE strips after 45 days of exposure to fungal isolates using the solid growth medium method, highlighting the isolates with the highest PE biodegradation. The spectra show changes in the chemical composition of **A** virgin and **B** non-virgin LDPE strips before pretreatment (original), after pretreatment (control), and after pretreatment followed by exposure to fungal isolates SP3MK, SP5INT, SP7MK, SP11INT, and SP17MK

**Fig. 10:**
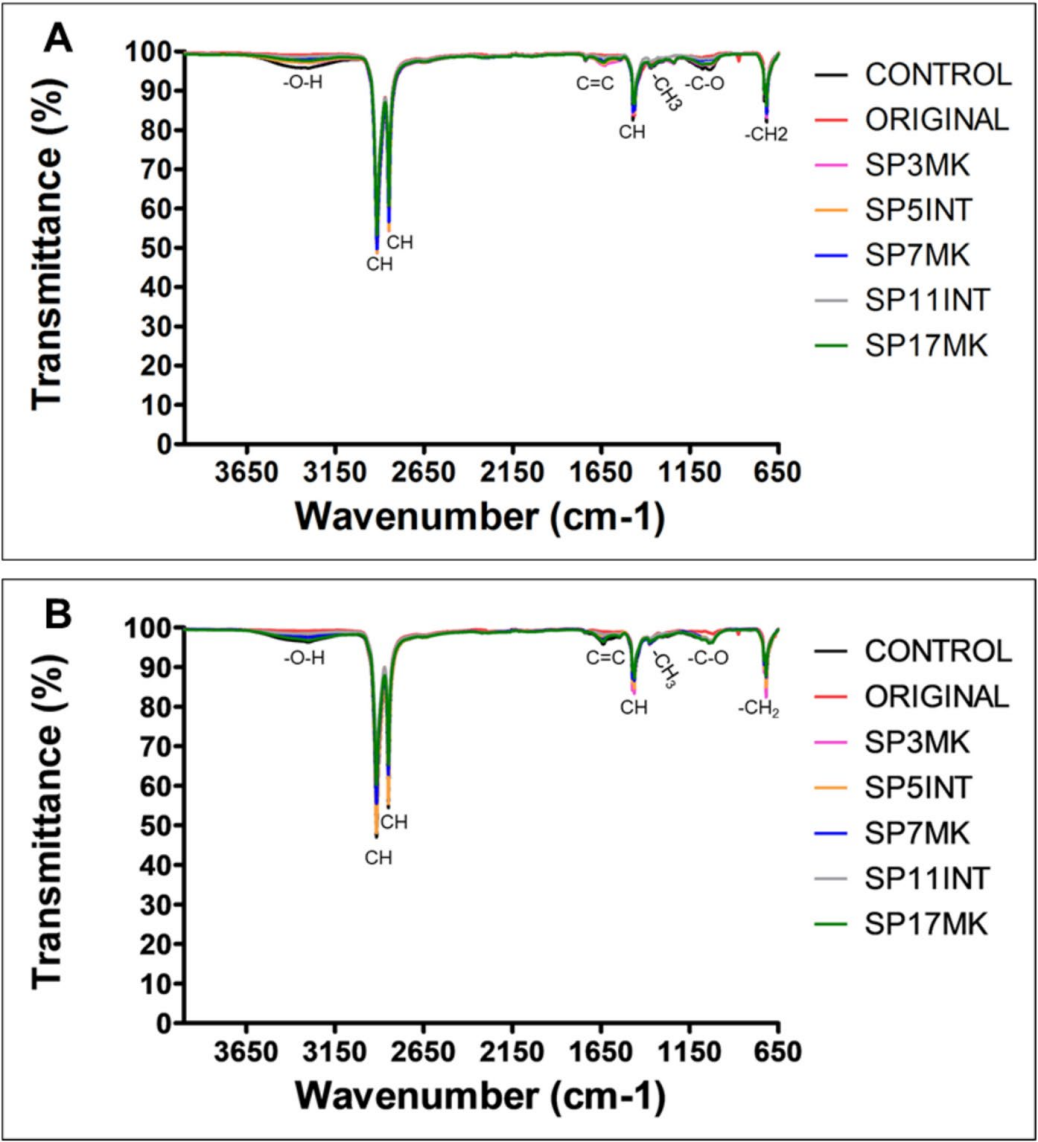
FTIR spectra of both virgin and non-virgin LDPE strips after 45 days of exposure to fungal isolates using the broth growth medium method, highlighting changes in chemical composition as an indication of biodegradation. The spectra show changes in the chemical composition of **A** virgin and **B** non-virgin LDPE strips before pretreatment (original), after pretreatment (control), and after pretreatment followed by exposure to fungal isolates SP3MK, SP5INT, SP7MK, SP11INT, and SP17MK

The FTIR was used to determine how fungal isolates affect polymeric functional groups and structure, as well as to detect the presence of chemical groups such as carbonyl, double bonds, and hydroxyl compounds. The extent of oxidation was determined using functional group indices, which show the band intensification of functional groups in relation to the C-H stretch of the spectra. The virgin and nonvirgin LDPE strips, as well as the control LDPE, were pretreated prior to the experiment to break the LDPE chemical bonds, allowing for easier fungal attack. The results were compared to pretreated control and untreated original LDPE strips. The results of virgin and nonvirgin LDPE after 45 days of incubation with fungal isolates are shown in Fig. 9 for the solid growth medium and in Figs. 10 for the LDPE that were exposed to fungal isolates using the broth medium, respectively.

### Virgin and non-virgin LDPE exposed on the solid growth medium

The most substantial chemical alterations in virgin LDPE were observed with SP5INT (Fig. 9A), as indicated by the emergence of a broad hydroxyl (-OH) absorption band at 3280 cm⁻^1^, a new alkene (C = C) band at 1646 cm⁻^1^, and significant reductions in the symmetric and asymmetric methylene (-CH) stretching vibrations at 2916 cm⁻^1^ and 2849 cm⁻^1^, respectively. These changes are characteristic of oxidative degradation and confirm a strong biochemical interaction between SP5INT and the polymer surface.

In non-virgin LDPE, isolates SP17MK, SP3MK, and SP5INT all induced notable spectral modifications (Fig. 9B). SP17MK exhibited the most pronounced effects, including attenuation of the -CH bending band at 1367 cm⁻^1^ and the appearance of additional bands corresponding to C = C and C–O functional groups. SP3MK also caused detectable changes, including the emergence of a band at 1635 cm⁻^1^ and reduction in the intensity of the 1367 cm⁻^1^ peak, suggesting moderate oxidative transformation of the polymer surface. While SP5INT elicited similar changes in non-virgin LDPE, the extent of modification was slightly reduced compared to its impact on virgin LDPE. In contrast, SP7MK induced only minimal chemical shifts, and SP11INT produced no discernible spectral changes in either polymer type, indicating negligible biochemical interaction with PE. Collectively, these findings demonstrate that SP5INT is the most effective in modifying virgin LDPE, whereas SP17MK, SP3MK, and SP5INT exhibit degradative potential toward non-virgin LDPE.

### LDPE exposed on the broth growth medium

Although substantial chemical composition changes were observed in LDPE strips after exposure to fungal isolates in solid medium, the same was not true for broth medium, where modifications were minimal or absent for most isolates (Fig. 10A,B). In the solid medium, isolates such as SP3MK and SP5INT induced clear structural alterations, including reduced intensity of -CH groups and the emergence of new functional bands. However, in the broth medium, fungal isolates caused far fewer chemical changes on both virgin and nonvirgin LDPE even after 45 days of incubation (Fig. 10A, B). While slight variations in peak ratios were detected particularly in key PE bands at 2916 cm⁻^1^ and 2849 cm⁻^1^ these were subtle compared to the pronounced transformations seen in the solid medium. Notably, the same isolates (SP3MK and SP5INT), which showed strong degradative effects in solid media, induced only modest reductions in CH bands in broth cultures (Fig. 10A, B).

### Functional group concentration changes in LDPE

In the solid medium, virgin LDPE strips treated with fungal isolates showed markedly lower carbonyl, double bond, and hydroxyl indices compared to untreated and pretreated controls (Table 4). Specifically, isolates SP5INT, SP3MK, and SP17MK reduced carbonyl indices to approximately 1.11–1.13, relative to the higher control values (~ 1.18–1.19). A similar pattern emerged in non-virgin LDPE, where SP5INT and SP17MK achieved the lowest carbonyl indices (0.94 and 0.88, respectively). Double bond indices largely mirrored this trend: in virgin LDPE, SP5INT exhibited the lowest value (0.87). Hydroxyl indices were highest in control samples, whereas treatment with SP5INT and SP17MK led to pronounced decreases (dropping to around 0.94 in virgin LDPE), reflecting microbial assimilation.

**Table 4:** Summary of FTIR-derived functional group indices and observed spectral changes in LDPE after incubation with fungal isolates

Isolate	Medium	PE type	Carbonyl index (~ 1700 cm⁻^1^)	Double bond index (~ 1646 cm⁻^1^)	Hydroxyl Index (~ 3280 cm⁻^1^)	New/Shifted Bands & Observed Changes
Control untreated	Solid	Virgin	1.15	1.18	1.9	–
Control pretreated	Solid	Virgin	1.16	1.19	2.14	–
SP3MK	Solid	Virgin	1.09 ↓	1.11 ↓	1.68 ↓	Emergence of 1635 cm⁻^1^ band; ↓ 1367 cm⁻^1^ peak
SP5INT	Solid	Virgin	0.87 ↓	0.87 ↓	0.94 ↓	Broad OH band at 3280 cm⁻^1^; new C = C band at 1646 cm⁻^1^; ↓ CH bands (2916 & 2849 cm⁻^1^)
SP17MK	Solid	Virgin	1.04 ↓	1.04 ↓	1.39 ↓	Attenuation of 1367 cm⁻^1^ peak; new C = C and C–O bands
SP7MK	Solid	Virgin	1.13 ↓	1.16 ↓	2.00	Minimal shifts
SP11INT	Solid	Virgin	1.12 ↓	1.14 ↓	1.81 ↓	No discernible changes
Control untreated	Solid	Nonvirgin	1.15	1.65	1.15	–
SP5INT	Solid	Nonvirgin	0.94 ↓	1.04 ↓	0.93 ↓	Emergence of 1635 cm⁻^1^ band; ↓ 1367 cm⁻^1^ peak
SP17MK	Solid	Nonvirgin	0.88 ↓	0.94 ↓	0.94 ↓	Attenuation of 1367 cm⁻^1^ peak; C = C and C–O bands
SP3MK	Solid	Nonvirgin	1.12 ↓	1.53 ↑	1.11 ↓	Emergence of 1635 cm⁻^1^; ↓ 1367 cm⁻^1^
SP7MK	Solid	Nonvirgin	1.14 ↓	1.68 ↑	1.12 ↓	Minimal chemical shifts
SP11INT	Solid	Nonvirgin	1.11 ↓	1.58 ↑	1.12 ↓	No discernible spectral changes
All isolates	Broth	Virgin & Nonvirgin	1.18	1.18	slight ↓	Only slight ↓ in CH bands; no major new bands

^a^(↓) indicates a decrease in functional group concentration compared to the pretreated control. (–) indicates no observable changes

Interestingly, for non-virgin LDPE, isolates SP3MK, SP7MK, and SP11INT instead induced increases in double bond indices (1.53–1.68) compared to the control (1.19). This rise suggests the formation of new C = C bonds associated with polymer chain scission and fragmentation. By contrast, SP5INT and SP17MK on non-virgin LDPE maintained lower double bond indices (1.04 and 0.94). In broth cultures, all fungal isolates produced only subtle chemical modifications: there was a slight reduction in methylene (–CH) band intensity and minimal formation of new functional groups. Functional group indices remained largely unchanged compared to controls.

Overall, these FTIR-based chemical changes demonstrated that solid-state conditions promote LDPE chemical structural changes. Among the tested fungi, SP5INT, SP3MK, and SP17MK consistently showed the strongest capacity to alter functional groups of LDPE strips (Table 4).

### Scanning electron microscopy (SEM) analysis

**Fig. 11:**
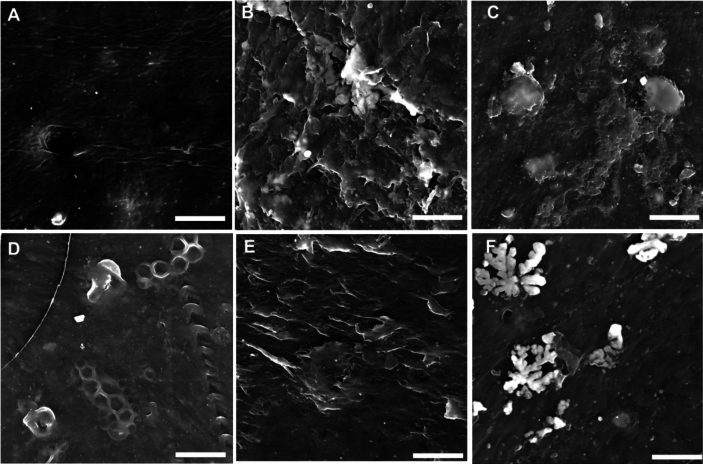
SEM micrographs (10 μm) of virgin LDPE strips after incubation for 45 days with fungal isolates in solid medium at room temperature. **A** The LDPE control was compared to (**B**–**F**) LDPE after exposure to fungal isolate SP3MK, SP5INT, SP7MK, SP11INT, and SP17MK. The fungal isolates caused varying degrees of PE surface corrosion and the formation of cracks and holes after incubation

**Fig. 12:**
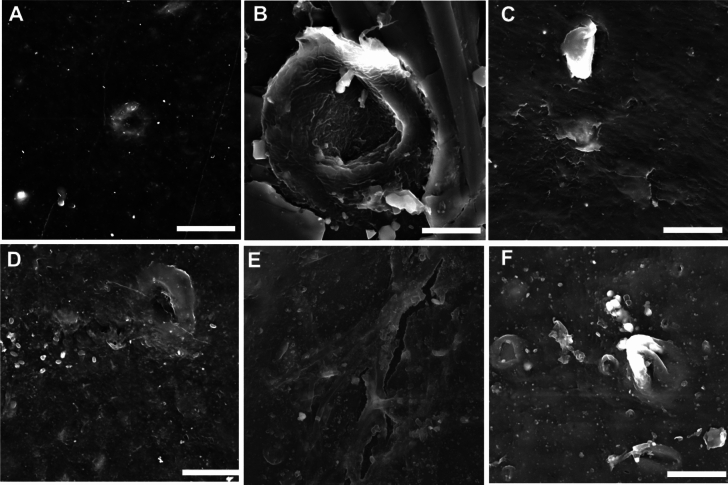
SEM micrographs (10 μm) of nonvirgin LDPE strips after incubation for 45 days with fungal isolates in solid medium at room temperature. **A** The PE control was compared to (**B**–**F**) PE after exposure to fungal isolate SP3MK, SP5INT, SP7MK, SP11INT, and SP17MK. The fungal isolates caused varying degrees of PE surface corrosion and the formation of cracks and holes after incubation

The presence of visible surface modifications after incubation with fungal isolates confirmed PE biodegradation, as shown in Fig. 11 for virgin LDPE and Fig. 12 for nonvirgin LDPE. The PE morphology and surface structure changed dramatically between the control and the fungal isolate-incubated PE after 45 days. The control PE for both virgin and nonvirgin LDPE was relatively smooth, with distinct heat reaction crinkles. Cracks, pits, and flaking on the surface of the PE indicated biodegradation by fungal isolates. After incubation, fungal isolates SP3MK, SP5INT, SP7MK, SP11INT, and SP17MK exhibited significant surface corrosion on virgin LDPE (Fig. 11).

These fungal isolates also caused significant surface corrosion, cracks, and holes in the nonvirgin LDPE, particularly SP3MK, SP5INT, SP11INT, and SP17MK (Fig. 12). Furthermore, fungal mycelia remained on the surface of the LDPE strips, indicating that the fungus had a strong adhesion to the PE surfaces. The adhered mycelia were visible on the virgin LDPE treated with SP7MK, SP11INT, and SP17MK (Fig. 12). Mycelia adhered to nonvirgin LDPE treated with SP7MK, and SP11INT (Fig. 12).

## Discussion

### Isolation, screening and identification of fungal isolates from landfill sites

Landfill environments harbour diverse fungi capable of metabolizing complex organic compounds, including synthetic polymers such PE (Adebayo and Obiekezie 2018). From the landfill samples, 18 fungal strains were isolated and screened for their ability to degrade PE. Five isolates viz*.*, *Engyodontium album* (SP3MK), *Arthrographis kalrae* (SP5INT), *Lecanicillium coprophilum* (SP7MK), *D. variabile* (SP11INT) and *Penicillium chrysogenum* (SP17MK) demonstrated significant biodegradation potential. Among these, SP5INT (*A. kalrae*), SP7MK (*L. coprophilum*), and *D. variabile* SP11INT represent novel isolates not previously reported in the context of PE degradation. This finding broadens the spectrum of fungal species with bioremediation potential, highlighting landfill sites as valuable sources of unexplored fungal biodiversity with plastic-degrading capabilities. SP3MK was initially misidentified morphologically but confirmed by molecular methods as *E. album*, underscoring the importance of molecular techniques for accurate fungal identification (Hoog 1978; Tsang et al. 2016).

### Biodegradation performance of selected fungal isolates on polyethylene

The ability of fungal isolates to colonize and degrade LDPE varied, with *P. chrysogenum* SP17MK and *E. album* SP3MK showing the highest degradation rates, achieving significant weight loss within 45 days. Notably, *A. kalrae* SP5INT and *L. coprophilum* SP7MK, though novel in this role, exhibited promising biodegradation activity, especially on non-virgin LDPE strips. Their performance indicates that these previously unreported fungal species may play important roles in plastic waste bioremediation. Isolate SP3MK exhibited greater degradation of non-virgin LDPE, achieving a 26% weight reduction after 45 days significantly faster than degradation rates reported for other fungi in longer timeframes (Samat et al. 2023). SP17MK was more effective on virgin LDPE. The susceptibility of non-virgin LDPE to fungal attack likely reflects its altered physicochemical properties resulting from prior recycling processes (Alzerreca et al. 2015; Zhou et al. 2014). Our results indicate that fungal isolate SP3MK exhibited higher biodegradation efficiency on non-virgin LDPE, whereas SP17MK was more effective on virgin LDPE strips. Notably, non-virgin LDPE appeared generally more susceptible to degradation by several fungal isolates compared to virgin LDPE. This increased susceptibility was supported by more pronounced chemical modifications detected in non-virgin LDPE following fungal incubation. Similar observations have been reported in previous studies. For example, Stapleton et al. (2023) found that polymers derived from recycled materials, such as polylactic acid, tend to degrade more rapidly, likely due to structural alterations introduced during recycling processes. Zheng et al. (2005) similarly noted that polymers retaining their original carbon backbone structures are generally more resistant to microbial attack than those previously exposed to environmental degradation.

The greater degradability of recycled plastics may be attributed to cumulative physical, chemical, and biological weathering processes such as exposure to UV light, heat, moisture, and microbial action that introduce reactive functional groups and modify polymer crystallinity and surface properties (Zhou et al. 2014). Additionally, Alzerreca et al. (2015) reported that recycled polyethylene exhibits reduced tensile strength compared to virgin LDPE, potentially enhancing fungal colonization and enzyme accessibility. However, to fully elucidate these effects, further research is needed to characterize the chemical and physical properties of non-virgin LDPE prior to treatment and to systematically assess their influence on fungal degradation efficiency.

Consistent with previous reports, an increase in pH was observed during fungal degradation assays, correlating with enzymatic breakdown of LDPE and release of alkaline by-products (Das and Kumar 2014; Nasrabadi et al. 2023). The isolates exhibiting the highest weight loss SP3MK, SP5INT, SP7MK, and SP17MK also caused significant pH shifts, reinforcing their biodegradation potential. This pH elevation likely reflects underlying biochemical processes: extracellular oxidative enzymes such as laccases and peroxidases introduce oxygen-containing functional groups (e.g., carbonyl and hydroxyl) into the PE backbone, promoting depolymerization. Subsequent microbial assimilation of these oxidized fragments and deamination reactions during amino acid catabolism can generate alkaline by-products such as ammonia or amines, contributing to medium alkalinization (Santo et al. 2013; Urbanek et al. 2018). Together, these processes may explain the observed association between PE weight loss and increased pH during fungal incubation.

### Confirmation of the ability of fungal isolates to biodegrade polyethylene

In this study, the biodegradation potential of fungal isolates was convincingly demonstrated through FTIR analysis of LDPE strips. FTIR spectra revealed significant chemical modifications in both pretreated virgin and non-virgin LDPE after incubation with the selected fungal isolates. Key indicators of biodegradation included the formation and alteration of functional groups such as carboxyl, hydroxyl (-OH), and double bonds (C = C). These spectral changes strongly support the enzymatic breakdown of the polymer structure by fungal activity. Notably, the intensity of these functional groups was higher in the control PE samples compared to those incubated with fungi after 45 days, indicating fungal-mediated consumption or transformation of these groups. This finding aligns with reports from a previous study (Samat et al. 2023), which observed that hydroxyl groups formed during pretreatment decreased after fungal incubation, suggesting active biodegradation rather than abiotic oxidation alone.

The current study observed the formation of -OH stretching bands after pretreatment and fungal exposure, consistent with prior research documenting new spectral bands attributed to polymer deterioration during microbial attack (Gong et al. 2023; Jung et al. 2018; Sangale et al. 2019). Specifically, after incubation with *A. kalrae* SP5INT and *P. chrysogenum* SP17MK, -CH bands were significantly reduced while broad, intense -OH bands appeared, indicating oxidative depolymerisation. Similar spectral transformations were previously reported (Jailawi et al. 2015) in bacterial PE degradation. The PE oxidation induced by abiotic pretreatments (UV, heat, chemical reagents), commonly referred to as auto-oxidation, is a critical step that facilitates fungal colonization by reducing hydrophobicity and enhancing surface reactivity (Hadad et al. 2005; Samat et al. 2023). This oxidation results in new functional groups that serve as points of enzymatic attack, initiating the biodegradation cascade. Our FTIR analysis revealed an increase in spectral bands between 1690 and 1615 cm⁻^1^, indicative of C = C double bond formation, a symbol of PE depolymerization and fragmentation (Eldin et al. 2022; Gong et al. 2023). In particular, *E. album* SP3MK showed a distinct peak at 1635 cm⁻^1^, confirming the introduction of C = C bonds, consistent with prior observations linking double bond formation to critical transition steps in polymer degradation (Eldin et al. 2022; Mohanan et al. 2020). These spectral signatures underscore enzymatic oxidation processes that contribute to the fragmentation and eventual mineralization of PE.

Interestingly, *A. kalrae* SP5INT exhibited intense bands between 1000–1150 cm⁻^1^, likely reflecting residual fungal proteins and carbohydrates, but also indicating active biochemical interactions with the PE surface (Lecellier et al. 2015). These findings are consistent with previous study (Muhonja et al. 2018), which linked such spectral changes to microbial degradation activities. The reduction of carbonyl concentration in LDPE strips incubated with *E. album* SP3MK, *A. kalrae* SP5INT, and *P. chrysogenum* SP17MK compared to controls further validates the biodegradation process, as carbonyl groups are often formed during abiotic oxidation but subsequently consumed or altered during microbial degradation (Samat et al. 2023; Sudhakar et al. 2007). This was more pronounced in non-virgin LDPE, suggesting that recycling-associated alterations in polymer structure may enhance fungal degradation efficiency. Scanning Electron Microscopy corroborated these chemical changes by revealing increasingly rough and pitted PE surfaces post-fungal treatment, consistent with enzymatic attack and physical disruption of the polymer matrix. The observed correlation between chemical changes, weight loss, pH shifts, and surface morphology strengthens the evidence for active PE biodegradation by the fungal isolates.

The markedly higher weight loss and more pronounced chemical modifications observed in solid-state cultures compared to broth cultures can be attributed to fundamental differences in fungal growth patterns and polymer–fungus interactions. Solid media likely facilitate tighter and more sustained hyphal penetration into the polymer matrix and stronger surface adhesion, thereby enhancing depolymerization and oxidation processes (Krishna 2005). These findings align with previous reports that solid-state fermentation environments favour more effective microbial colonization, localized enzyme production, and bio-fragmentation of recalcitrant polymers, ultimately leading to enhanced biodegradation performance (Pandey et al. 2000). In contrast, submerged broth cultures tend to promote dispersed or pelleted mycelial growth, which limits persistent surface contact with the hydrophobic PE strips and may reduce the effectiveness of enzymatic degradation (Awasthi et al. 2017; Sen and Raut 2015). It is worth noting that previous research has shown that extending the plastic immersion period and applying longer or more intensive pretreatment can further improve biodegradation in broth media by increasing the hydrophilicity of the polymer surface, thereby facilitating fungal attachment and enzyme access (Awasthi et al. 2017). Collectively, these findings highlight the critical role of physical contact and surface properties in determining the effectiveness of polyethylene biodegradation by fungi.

### Novel fungal isolates with potential for PE biodegradation

To our knowledge, the fungal isolates *A. kalrae* SP5INT, *L. coprophilum* SP7MK, and *D. variabile* SP11INT have not been previously documented for their polyethylene biodegradation capabilities. Our findings position these isolates as promising candidates for further exploration in plastic waste bioremediation. *L. coprophilum* SP7MK, traditionally recognized for entomopathogenic traits, demonstrated notable PE degradation activity, suggesting unexplored enzymatic capacities. Similarly, *D. variabile* SP11INT, known for producing laccase enzymes often involved in lignin and polymer degradation, showed modest weight loss but clear mycelial adherence to PE surfaces, implying biodegradation potential. These novel isolates consistently induced the formation of new functional groups such as carbonyls, aldehydes, ketones on the LDPE surface as detected by FTIR, suggesting active enzymatic modification of the polymer. Although the precise biochemical pathways remain to be elucidated, these initial results warrant deeper investigation of their enzymatic machinery and long-term degradation potential.

## Conclusion

This study demonstrates the potential of landfill-derived fungi to biodegrade LDPE, highlighting both known and novel fungal species as promising candidates for bioremediation. Among eighteen isolates initially screened, five species *Engyodontium album* (SP3MK), *Penicillium chrysogenum* (SP17MK), *Arthrographis kalrae* (SP5INT), *Lecanicillium coprophilum* (SP7MK), and *Didymosphaeria variabile* (SP11INT) showed measurable LDPE degradation, confirmed through gravimetric analysis, FTIR spectroscopy, and SEM imaging. Notably, *A. kalrae*, *L. coprophilum*, and *D. variabile* have not been previously reported for polyethylene degradation, expanding the known diversity of fungal species capable of attacking recalcitrant polymers. The study also revealed that biodegradation efficiency varied by plastic type and growth conditions. Nonvirgin LDPE, likely possessing higher surface oxidation from prior recycling, was generally more susceptible to fungal attack. Furthermore, solid-state cultures promoted significantly higher weight loss and more pronounced chemical modifications compared to submerged broth cultures, underscoring the importance of direct hyphal contact and localized enzyme activity in polymer degradation. Overall, this work contributes novel data on the biodegradation potential of landfill-adapted fungi, provides a scientific foundation for their further study, and supports the development of sustainable, biologically based strategies to mitigate plastic pollution.

Despite these insights, the study has limitations. The experiments were conducted under controlled laboratory conditions over a relatively short period (45 days), which may not fully capture long-term or environmental dynamics of fungal plastic degradation. In addition, although FTIR and SEM analyses provided strong evidence of polymer oxidation and surface erosion, the precise enzymatic pathways and metabolic intermediates involved remain to be characterized. Future research should explore longer incubation periods, enzyme assays, and genetic profiling to elucidate the biochemical mechanisms underlying fungal polyethylene degradation. Moreover, scaling these findings for environmental or industrial application will require pilot studies under real landfill or composting conditions.

## Data Availability

Data is provided within the manuscript.
